# Genome biology of long non-coding RNAs in humans: A virtual karyotype

**DOI:** 10.1016/j.csbj.2025.01.026

**Published:** 2025-01-31

**Authors:** Alessandro Palma, Giulia Buonaiuto, Monica Ballarino, Pietro Laneve

**Affiliations:** a Department of Biology and Biotechnologies “Charles Darwin”, Sapienza University of Rome, Piazzale Aldo Moro 5, Rome 00185, Italy; b Institute of Molecular Biology and Pathology, National Research Council of Italy, Piazzale Aldo Moro 7, Rome 00185, Italy

**Keywords:** Long non-coding RNA, Genomics, Chromosomes, SNPs, Functional genomics

## Abstract

Long non-coding RNAs (lncRNAs) represent a groundbreaking class of RNA molecules that exert regulatory functions with remarkable tissue and cellular specificity. Although the identification of functionally significant lncRNAs is increasing, a comprehensive profiling of their genomic features remains elusive. Here, we present a detailed overview of the distribution of lncRNA genes across human chromosomes and describe key RNA features—what we refer to as a “virtual lncRNA karyotype”—that provide insights into their biosynthesis and function. To achieve this, we leveraged existing human annotation files to construct a statistical genomic portrait of lncRNAs in comparison with protein-coding genes (PCGs). We found that lncRNAs are unevenly distributed across chromosomes and identified regions of high lncRNA density on chromosomes 18, 13, and X, which overlap with PCG-rich regions. Additionally, we observed that lncRNAs generally exhibit shorter gene lengths and fewer splicing variants compared to protein-coding transcripts, with a subset displaying pronounced clustering patterns that may indicate functional relevance. Finally, we identified several clinically associated and experimentally validated SNPs impacting lncRNA genes (lncGs). Overall, this study provides a foundational reference for exploring the non-coding genome, offering new insights into the genomic characteristics of lncRNAs. These findings may enhance our understanding of their biological significance and potential roles in disease.

## Graphical Abstract

**Figure:**
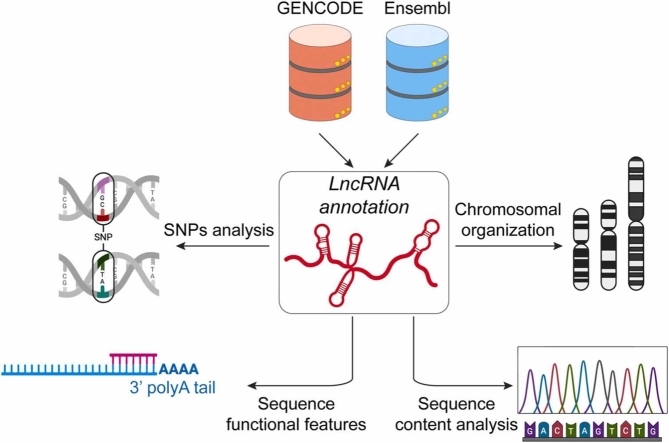

## 1 Introduction

Long non-coding RNAs (lncRNAs) emerged in the early to mid-2000s as surprising evidence of the genomes’ transcriptional pervasiveness. Initially considered junk material, their importance as precise spatiotemporal tuners of gene expression has become increasingly recognized with the accumulation of functional data, reshaping our mechanistic interpretation of the genetic information flow [33]. The lncRNA attributes, including their high expression specificity and ability to scaffold chromatin, RNA, and proteins underlie their influence on cellular identity and activity [43], [48].

Additionally, their propensity for rapid evolution contributes to biological complexity. The extent of this potential underscores the necessity of detailed annotations to provide essential context and information for the functional descriptions of lncRNA genes (lncG), as well as their genetic variations that can impact health and disease. However, lncRNAs remain relatively understudied from this standpoint [2]. In particular, the lack of comprehensive structural and functional annotations hinders the investigation of their roles in cellular processes and functional enrichment studies derived from increasingly widespread next-generation sequencing data.

As the primary structure of lncRNAs shapes their biological properties and binding to specific partners, many computational tools have been developed for its analysis (as reviewed in [6]). Notably, sequence features in introns retained after splicing completion are of increasing interest, as these have been shown to influence the scaffolding activities and the specific binding of lncRNAs to macromolecular interactors [12], [13], [14], [39], [51].

LncRNA classification was initially based on the lack of coding potential [29], although recent discoveries have revealed that certain lncRNAs can encode functional micro-peptides (reviewed in [22], [3], [36]). Furthermore, a recent consensus proposed classifying these transcripts as being longer than 500 nt, to exclude a range of small RNAs (e.g., RNA polymerase III transcripts, small nuclear RNAs (snRNAs), and intron-derived small nucleolar RNAs (snoRNA) that are 50–500 nucleotides in length [33]. Overall, it emerges that the classification of lncRNAs is not always clear-cut due to their functional heterogeneity, necessitating a deeper study of their genomic organization to gain a more comprehensive understanding of this RNA class.

In the human genome, protein-coding genes (PCGs) and lncGs are distributed along nearly 3.2 billion base pairs of DNA, arranged across the 46 chromosomes in a precise manner. The genomic arrangement of lncGs has not been extensively studied yet, but it is plausible that it could affect the role of lncRNAs as regulators of gene expression. To address this issue, we examined the genomic organization of lncGs in the human genome using annotation data from genomic databases including GENCODE [16], Ensembl [32] and dbSNP [42], and captured genomic features like chromosomal distribution, nucleotide content, and point mutations with possible roles in lncRNA biology.

## 2 Results

### 2.1 Distribution of lncGs along the genome

**Fig. 1:**
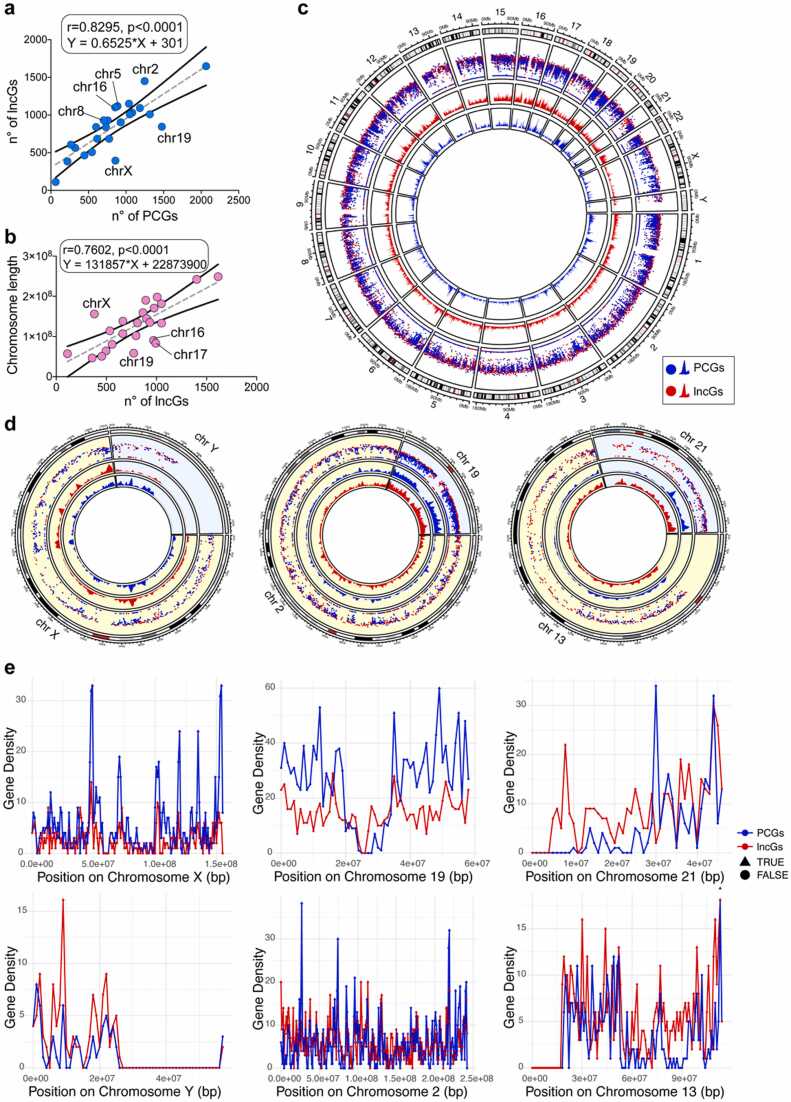
Chromosomal distribution of protein-coding and long non-coding RNA genes. Correlation between the number of long non-coding RNA (lncGs) and protein-coding genes (PCGs) (a) or chromosome length (b) across chromosomes. (c) Rainfall plot showing the distribution of human PCGs (blue) and lncGs (red) across chromosomes. Blue and red dots represent single PCGs and lncGs, respectively. Blue and red distributions correspond to PCGs and lncGs, respectively. (d) Insets on chromosomes X, Y, 2, 19, 13 and 21. Dots represent individual genes, while peaks represent distributions within a 1 Megabase window. Chromosomes were randomly paired together. (e) Peaks quantification of the density of lncGs and PCGs in the corresponding (insets) chromosomes (see Methods for the quantification methodology). "TRUE peaks" and represented as triangles, while “FALSE peaks” were represented as circles (see Methods for the definition).

To update previous contributions [11] and provide possible insights into the functional significance of lncGs, we analyzed their distribution across the human genome. In line with the FANTOM5 project [1], we queried the current GENCODE human catalog (version 46), which lists a comparable number of long non-coding (20,310) and protein-coding (20,065) genes, although with a different sequence coverage (614,682,854 nt for lncGs and 1380,521,881 nt for PCGs, respectively). Despite the positive correlation (R=0.68), the distribution of lncGs and PCGs across chromosomes is non-uniform (Suppl. Fig. 1a). Specifically, we found that chromosomes 19, 11, and X exhibit significantly more PCGs, whereas chromosomes 2, 5, and 8 display a higher number of lncGs (Fig. 1a). Chromosome 1 harbors the highest absolute number of lncGs (1649), followed by chromosome 2 (1451), chromosome 12 (1150), and chromosome 5 (1118) (Supp. Fig. 1a and Suppl. Table 1). In contrast, the Y chromosome contains the fewest lncGs (112), which are restricted to its short arm and to a proximal region of the long arm. Additionally, chromosome length also appears to be correlated with lncG abundance (R=0.58), albeit with some exceptions. For instance, chromosome X contains fewer lncGs than expected based on its large size (Fig. 1b), while the smaller metacentric and submetacentric chromosomes 16, 17, and 19 exhibit a higher number of lncGs relative to their length. Notably, chromosome 16 stands out as the second most lncG-dense chromosome, with approximately 10.5 lncGs per megabase (Mb).

Furthermore, an analysis of the overall lncG densities (calculated as the number of lncGs per 1 Mb for each chromosome), revealed high densities and local peaks of lncGs or PCGs in certain chromosomal regions (Fig. 1c, d).

Notable examples of lncG local peaks are evident on chromosome 18. Conversely, a higher density of PCGs with marked local peaks is found on chromosomes 13 and X (Fig. 1c, d). Other chromosomes show comparable densities between coding and long non-coding RNA genes. For instance, chromosome 19 exhibits a lncGs density (845, corresponding to 14.42 per Megabase (Mb)) that partially reflects that of coding genes. PCGs are extremely numerous on chromosome 19 as well (1477 genes, 25.2 genes/Mb) (Fig. 1a), in line with chromosome 19 being considered the most gene-dense human one [19]. A unique case is represented by chromosome 21, which exhibits a distinct separation between a region enriched in lncGs, on its short arm and the proximal part of the long arm, and a region abundant in PCGs, the latter situated in the distal part of its long arm (Fig. 1c, d). Another key feature is present on Y chromosome, which displays a higher lncGs’ density compared to PCGs. Indeed, peak quantification on these chromosomes (Fig. 1e) clearly shows an uneven distribution of lncGs, identifying distinct chromosome areas that are particularly enriched in lncGs. These peaks are regions where the gene density exceeds a certain threshold, indicating areas of unusually high concentration of either lncGs or PCGs.

Collectively, these findings indicate that lncGs are almost unevenly distributed throughout the human genome, with specific chromosomes or chromosomal regions that appear enriched in either lncGs or PCGs. The presence of local peaks of lncGs along chromosomes, in particular on chromosomes Y and 21, hints at positional clustering, which could potentially impact lncRNA expression and function.

### 2.2 Long non-coding genes: about size and nucleotide content

**Fig. 2:**
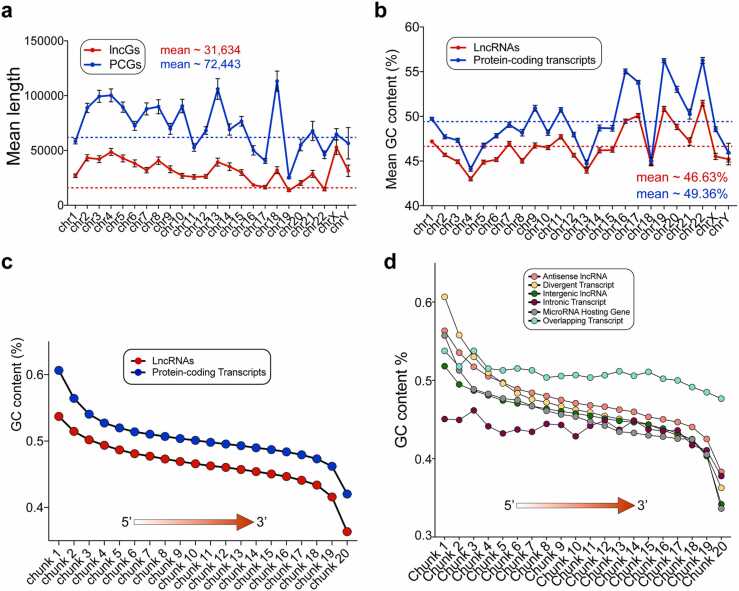
Length and GC content of long non-coding and protein-coding genes and transcripts. (a) Average length and (b) GC content of long non-coding and protein-coding transcripts across chromosomes. (c) GC skew of coding and long non-coding transcripts divided by 20 chunks proportional to each transcript length from 5’ to 3’. (d) GC skew of long non-coding transcripts for the distinct lncRNA subtypes. The definition and computation of the chunks are the same for both panels c and d (see Methods).

To delve into more sequence-specific features, we analyzed the lengths of PCGs and lncGs. Our findings revealed a significant variability in lncG length, averaging 31,634 nucleotides (nt), which was quite dependent on chromosome size, and tended to be shorter than PCGs averaging 72,443 nt in length (Fig. 2a). Shorter lncGs were typically located on smaller chromosomes such as 16, 17, 19, and 22, while longer lncGs were found on larger ones such as chromosomes 4 and X (Fig. 2a), with few exceptions. For instance, despite chromosome Y being the third smallest human chromosome, it contains 112 lncGs (mean length: 31,439 nt). Their length is comparable to the average length of lncGs across all chromosomes. Conversely, chromosome 1, the largest human chromosome spanning 248,956,422 nt, harbors the highest number of lncGs, totaling 1649. However, the average length of these lncGs is 26,902.7 nt. This value falls significantly below the overall mean length of lncG from all chromosomes.

As for the transcript sequences, we examined all the long non-coding transcripts that are annotated on GENCODE and observed that lncRNAs exhibited substantial heterogeneity, boasting a mean length of 1319 nt and a median length of 965 nt. Notably, the length distribution of lncRNAs displays three discernible peaks (Suppl. Fig. 1b) at 600, 800, and 1500 nucleotides. This distribution hints at the possibility for classifying these transcripts into three different subcategories, based on their length. Moreover, it is worth noting that most of lncG sequences contain introns.

We then zoomed in the nucleotide composition. Regions of DNA characterized by high guanine and cytosine contents (GC-rich regions) are recognized for their significance in the formation of stable structures, thereby influencing histone deposition and genome functionality [54]. These regions often exhibit a high density of CpG dinucleotides, serving as regulatory islands for a majority of human genes [25]. GC skew is frequently linked to unmethylated human CpG island promoters [18], and their transcription can instigate the formation of R-loops stabilizing the nascent RNA at its DNA locus and generating GC-rich RNA:DNA hybrids [23]. The presence of these structures potentially underlie the role of lncRNAs as epigenetic regulators, as already shown for selected species [17], [34], [4], [9].

Earlier analyses have indicated that lncRNAs typically show lower GC content compared to protein-coding transcripts, with exons generally bearing a higher GC content than introns [20], [38]. However, this distinction should be interpreted cautiously and scrutinized for potential analytical biases, as some lncRNAs retain introns in their functional form [12]. The average GC content of the human genome is approximately 41 % [10], [28], [37], with chromosomes 19 and 22 characterized by the highest percentage (47.94 % and 47 %, respectively). When assessing the GC content of long non-coding transcripts and PCG sequences across different chromosomes, we noted an overall mean of 46.6 % for lncRNAs, reaching values as high as 49.4 % for protein-coding transcripts (Fig. 2b). When looking at lncRNAs individually in each chromosome, chromosomes 16, 17, 19, 20, and 22 stood out with the highest GC content, surpassing the 50 % threshold, while chromosomes 4 and 13 displayed relatively lower GC content at 43 % and 43.9 %, respectively. To investigate whether this GC content was maintained in the fully spliced form of lncRNAs, we examined the positional effect of GC skew. Employing a 10 nt sliding window, we found that GC content was much higher near the 5’ end of the sequence for both coding and non-coding transcripts, with the former bearing a higher GC content. In contrast, GC content significantly decreased toward the 3′ end, reaching values as low as 36.3 % for lncRNAs (Fig. 2c). As for protein-coding genes, the highest content of GC peaks at the 5′ end may be associated either with the transcriptional regulation or the nuclear export of lncRNAs. The study of the evolutionary origin, maintenance, and distribution of these regions across lncRNA genes is crucial and will help to understand alterations in lncRNA expression in pathological contexts. We noted a tendency for GC content to be lower in nuclear-localized lncRNAs when compared to cytoplasmic ones (nuclear lncRNAs mean=49.1 %, cytoplasmic lncRNAs mean=50. 4 %, Suppl. fig. 1c). However, the observed difference is not statistically significant suggesting that the subcellular localization of these lncRNAs seems to be generally independent of GC content (ANOVA test adjusted p-value=0.15). Potential bias on this observation may derive from the scarcity of genomic data, which highlights the necessity of a more robust lncRNA annotation.

#### 2.2.1 Functional classification of lncG types

Over the years, the heterogeneity of lncRNA classes has led to their classification using various parameters, including their genomics [41] or other functional features [29]. Here, we introduce the designation "Novel transcripts" for lncGs identified solely by the Ensembl ID. Moreover, we attribute "Name assigned lncRNAs" to those lncGs with a given arbitrary gene name and documented functional characterization, such as *MALAT1* and *XIST* (Table 1).

**Table 1:** LncG classification. LncG subtypes are reported together with a short description, the prefix or suffix used in their gene name, and their numerosity in the human genome.

**LncRNA subtypes**	**Description**	**Nomenclature characteristic**	**Abundance in human genome**
Novel transcripts*	Newly identified lncRNAs	ENSG	14,320
Name assigned lncRNAs*	LncGs that have been attributed an arbitrary gene name	none	1040
Antisense LncRNAs	LncGs that totally or partially overlap the genomic sequence of a protein-coding gene on the opposite strand	-AS1 -AS2 …	1848
Divergent Transcripts	LncGs transcribed from a bidirectional promoter in the opposite direction to a protein-coding gene	-DT	600
Intergenic LncRNAs	LncGs not overlapping a protein-coding gene, not sharing a bidirectional promoter with a protein-coding gene, and not hosting microRNA or snoRNA genes	LINC	2274
Intronic Transcripts	LncGs transcribed entirely from an intron of a protein-coding gene on the same strand	-IT1 -IT2 …	127
MicroRNA Hosting Genes	LncGs hosting microRNA genes	-HG1 -HG2 …	86
Overlapping Transcripts	LncGs overlapping protein-coding genes on the same strand	-OT1 -OT2 …	15

(*) These two lncRNA subclasses include transcripts that may belong to other categories, as they have been recently identified or assigned an arbitrary gene name, which makes their classification ambiguous.

In line with the lack of official gene names or functional classifications for the majority of lncRNAs, we categorized a substantial portion of lncGs (approximately 70.5 %) as novel. Accordingly, only 5.1 % of lncGs are categorized as “Name assigned”.

The “Long intergenic” (LINC) class makes up approximately 11.2 % of all lncGs and is associated with a lower GC content compared to other lncRNA subtypes, particularly evident in their 3′ end (Fig. 2d). The category “Overlapping transcripts” includes lncRNAs predominantly located on chromosome 3 (Suppl. Fig. 1d). A notable characteristic of this group is the variable length of genes with a mean of 61,152.1333 nt (SEM= 31,091.165). Additionally, these transcripts exhibit a relatively constant GC content along their sequence, ranging from a maximum of 54 % to a minimum of 48 %, which could relate to their intrinsic characteristic of overlapping a PCGs (Fig. 2d). MicroRNA hosting genes are predominantly located on chromosomes 21 (24 %) and 20 (15.1 %), while they are absent on chromosomes 3 and Y. These genes are also the longest ones among lncGs, with an average length of 118,660.023 nt, and their transcripts display the lowest GC content at the 3′ end compared to all other lncRNAs (33.6 %) (Fig. 2d). Finally, “divergent transcripts” represent the lncRNA class with the most pronounced difference in GC content between their extremities, ranging from 60.7 % at the 5′ end to 36.3 % at the 3′ end (Fig. 2d). Since these transcripts are transcribed in the opposite direction to PCGs, it remains to be determined whether the skewed GC content is functionally related to their bidirectionality.

It is worth noting that this classification is not exhaustive, and it is influenced by several factors, including the diverse and heterogeneous features considered, the substantial portion of unannotated transcripts, and the potential misclassification of lncRNAs that have already been characterized (name-assigned lncRNAs). For instance, although valuable, the “Novel” and “Name assigned” categories had to be excluded from GC content distribution analyses as they may include transcripts that belong to other subclasses. Nevertheless, our efforts serve to highlight challenges in classification and to refine previous quantification across lncGs based on their genomic, structural and functional characteristics.

### 2.3 Regulatory and functional elements of lncGs

Most of the lncRNAs are cleaved and polyadenylated at their 3’ends, like the majority of the RNA polymerase II-transcribed genes. Others, instead, are stabilized by alternative mechanisms, including RNase P cleavage [47], [8], or processed on both ends by the snoRNA machinery [49]. Upon analyzing the presence of canonical polyadenylation (polyA) sites (CA) and polyA signals (AATAAA) within the genomic sequences of lncGs, we discovered that nearly 28 % (5587) of lncGs exhibit the presence of either a polyA site or a polyA signal. This relatively low percentage aligns with other reports indicating that lncRNAs are predominantly non-polyadenylated [40]. The mean distance between the polyA site and the polyA signal was 20.7 nt and 20.3 nt for protein-coding and lncG sequences respectively (Suppl. Fig. 1e), indicating no evident differences between the two classes in terms of the organization of polyadenylation elements within their genomic sequences.

As polyA regulates mRNA export to cytoplasm and translation, we analyzed the presence of canonical polyA sites and signals in lncGs in relation to the subcellular localization of their related transcripts. By querying the "RNAlocate" tool (http://www.rna-society.org/rnalocate/) database, which includes 589 annotated lncRNAs along with their subcellular localization, we found no significant correlation between the presence/absence of polyA signals and the localization of specific lncRNAs into nuclear or cytoplasmic compartments (Fisher exact test p-value=0.126).

Similar to PCGs, lncGs are composed of one or more exons and introns within their genomic sequence. Some lncGs can be transcribed into different isoforms due to alternative splicing, variations in 5’ or 3′-ends, exon number, or the presence of introns that are not removed by the splicing machinery. Most PCGs are polyexonic, with only 4.5 % being monoexonic. On average, they consist of 70 exons (median=34) resulting in a total of 171,410 distinct annotated transcripts (Table 2). Each gene encodes an average of 8.5 diverse RNA isoforms (median=6), produced by alternative splicing. We found *RBFOX1* (RNA Binding Fox-1 Homolog 1) to be the longest PCG (2473,539 nt), coding for 40 different splicing variants.

**Table 2:** Genomic elements of protein-coding and non-coding genes, including transcript isoforms and exon numbers.

	**Protein-coding genes/transcripts**	**Long non-coding genes/transcripts**
Number of genes	20,065	20,310
Number of transcripts	171,411	59,928
Mean number of exons per gene	70.1	11.6
Median number exons per gene	34	3
Number of mono-exonic genes	973	3173
Mean number of exons per transcript	8.2	3.8
Median number of exons per transcript	6	3
Mean number of transcripts per gene	8.5	3.1
Median number of transcripts per gene	6	1

From the lncRNA perspective, the longest gene is annotated as *NRXN1-DT*, a divergent transcript with a genomic length of 1375,317 nt. This lncG is located on the positive strand of chromosome 2 and is transcribed in its full length into a 3939 nt monoexonic RNA. Considering all lncGs, 15.6 % of them are monoexonic while the others exhibit a varying number of exons, with a mean number of exons in the encoded transcripts that is lower than that of protein-coding transcripts (3.8 exons for lncRNAs, 8.2 for protein-coding transcripts). The lncRNA with the highest number of exons is *SNHG14*, a spliced paternally imprinted lncRNA located in the Prader-Willy critical region, comprising 59 different exon combinations and contributing to the transcription of 142 different splicing variants. However, the lncRNA producing the highest number of splicing variants is the antisense *PCBP1-AS1* (the second in rank for number of exons) with 296 annotated transcripts. In total, considering all lncGs, they possess a mean of 3.1 distinct splicing variants, contributing to a total of 59,928 long non-coding transcripts.

Taken together, these results underscore potentially crucial functional features of lncRNAs that distinguish them from PCGs (Table 2). These features include polyA, as well as a lower number of exons within their sequence, and a reduced number of splicing variants.

### 2.4 Nucleotide variations in long non-coding RNA genomic sequences

Nucleotide variation can significantly impact gene functionality [21], [27], including lncGs [15]. Mutations occurring in promoter regions or splicing sites could profoundly affect gene expression, while mutations in regulatory binding regions (such as those involved in transcription factor or RNA-binding protein interactions) could impact the ability of the gene product to interact with RNAs, DNA sequences, and proteins.

**Fig. 3:**
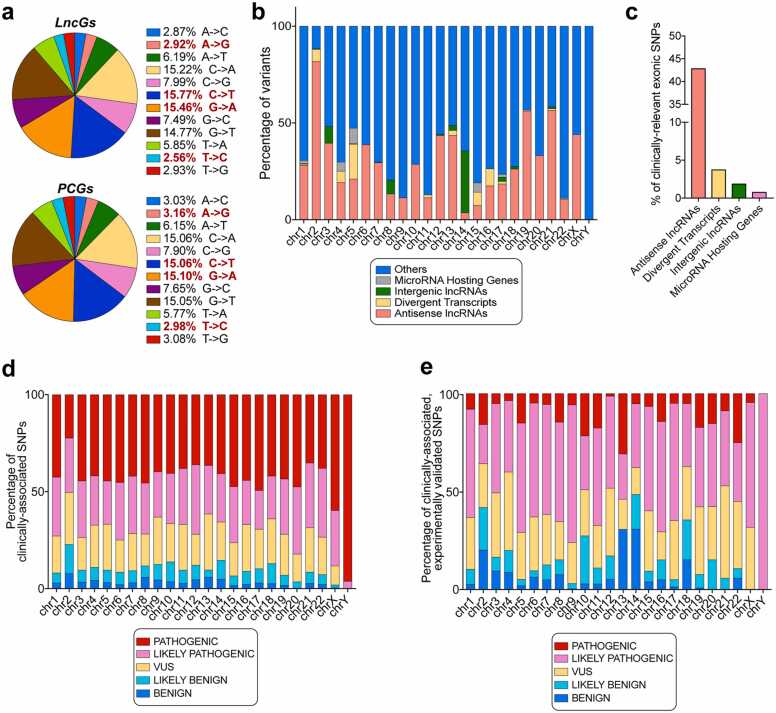
Analysis of SNPs across long non-coding genes. (a) Piechart showing the percentages of transitions and transversions in lncGs (upper piechart) and PCGs (lower piechart). Transitions are bolded and red colored. (b) Percentages of variants on the distinct chromosomes divided by long non-coding subtype. (c) Barplot showing the percentage of clinically associated SNPs in the distinct long non-coding RNA subtypes. (d) Barplot showing the percentage of clinically associated variants in lncGs, grouped by pathogenicity across human chromosomes. (e) Barplot showing the percentage of clinically associated and experimentally validated SNPs across human chromosomes.

Our analysis of the human single nucleotide variations (SNVs) cataloged in dbSNP [42], identified approximately 130 million single nucleotide polymorphisms (SNPs) associated with lncG regions. Among clinically annotated single nucleotide substitutions, transversions were the most common, accounting for 63.3 % of all SNPs in lncGs and 63.7 % in protein-coding genes (PCGs) (Fig. 3a). We observed no significant differences in the frequency of SNPs between lncGs and PCGs.

According to Ensembl, a number of 14,303 variants in lncGs are classified as clinically relevant, with 36.4 % labeled as pathogenic, 27.4 % as likely pathogenic, and 22.1 % as variants of uncertain significance (VUS). We then analyzed lncRNA subtypes to determine those more susceptible to clinically associated mutations (Fig. 3b). Intronic and overlapping transcripts showed no clinically associated variants. However, two categories stood out for their susceptibility to SNPs, the antisense RNAs and the novel transcripts. Antisense transcripts were highly enriched on chromosome 2, comprising the majority of variants observed in this region. By contrast, the Y chromosome exhibited only variants associated with name-assigned lncRNAs.

Notably, divergent transcripts were predominantly affected on chromosome 2, accounting for 56 % of total mutations in this RNA class. Conversely, variants in long intergenic non-coding RNAs (LINCs) were primarily distributed across chromosomes 3, 8, and 14, even though LINCs are predominantly located on chromosome 13. MicroRNA-hosting genes appeared to be the least affected by SNPs, constituting just 0.74 % of all SNPs, with most of these variants located on chromosomes 1, 4, 5, 15, and 17 (Fig. 3c). Overall, our results indicate that the differences between the observed and expected SNP distributions across long non-coding RNA classes for all chromosomes are highly statistically significant (chi-square test p-value < 0.0001 for all chromosomes, see Suppl. Table 2). When examining the pathogenicity of SNPs across individual chromosomes, similar patterns emerged (Fig. 3d). The Y chromosome stood out, containing 26 out of 27 pathogenic variants, along with one likely pathogenic variant. Chromosome 2 had the lowest percentage of pathogenic variants. Following the Y chromosome, the X chromosome exhibited one of the highest proportions of pathogenic variants (59.7 %). This chromosome is of particular significance due to the presence of XIST, a well-characterized lncRNA responsible for X-chromosome inactivation in female mammals [31]. Interestingly, while chromosome 2 had the highest absolute number of clinically associated variants (n = 4552), these represent 31.8 % of all variants in lncGs. Altogether, these findings suggest that lncRNA genomic regions are characterized by an uneven distribution of SNPs, with clinically significant variants being concentrated on distinct chromosomes, each exhibiting different levels of pathogenicity. Given the significant role of lncRNAs in human diseases, we explored the lncRNASNP v3 database (https://gong_lab.hzau.edu.cn/lncRNASNP3#!/) to investigate experimentally validated nucleotide variations. Our focus was on variants impacting lncRNAs that are both clinically associated and experimentally confirmed. This analysis revealed a total of 8016 variants, with varying degrees of pathogenicity across chromosomes (Fig. 3e). Notably, most of these variants were classified as either likely pathogenic or of uncertain significance. This classification may reflect the challenge in experimentally defining the impact of single nucleotide variants on lncRNA functions compared to protein-coding genes. Further research is needed to elucidate how these variants influence lncRNA function and whether they are linked to specific human pathological phenotypes.

## 3 Discussion

Compared to mRNAs, where the genetic code guides functional inferences, lncRNAs are more puzzling to characterize due to their complex transcriptome and processing, lack of evolutionary constraints, and limited structural/functional correlations. The study is even more complicated by the fact that the classification of these species relies on multiple features, including sub-cellular localization, position relative to coding genes, or functional roles. Therefore, the continuous update of lncRNA annotation becomes crucial for fully exploiting the potential of the noncoding genome and achieving significant advancements in biomedical research. Through this work, we delineate the current genomic landscape of lncGs, providing a comprehensive view of their structure, organization, and distribution across human chromosomes, effectively creating a virtual lncRNA karyotype. This reference framework can serve as a baseline for identifying physiological benchmarks, offering insights into their potential biosynthesis and function. Furthermore, it can be used to assess chromosomal aberrations such as copy number variations, fusions, and large structural rearrangements that may affect lncRNAs in pathological conditions.

By capturing and processing data from the available human gene set in GENCODE (see **Methods**), we show a snapshot of the current lncGs numerosity. Their abundance is comparable to that of protein-coding genes (PCGs), with around 20,000 instances and an average length of about 31,600 nt, which is consistently shorter than PCGs (72,400 nt). In addition, we provide an updated version of the genomic distribution for both PCGs and lncGs, describing their non-uniform arrangement in different chromosomes and highlighting distinct chromosomal regions with high lncRNA density. Notably, chromosome 2 was found to harbor the highest density of lncGs and is more affected by clinically relevant SNPs, although they appear to be less pathogenic compared to those on other chromosomes. As telomere-telomere fusion from ape chromosomes was proposed to give rise to the human chromosome 2 [26], the high lncG density can provide new hypotheses for understanding chromosome evolution. Additionally, we identified positional clustering of lncGs, particularly on chromosomes 21 and Y. This observation may suggest the existence of genomic loci dedicated to lncRNAs, which could be further and specifically focused through quantitative gene expression analyses to assess whether this positional clustering underlies functional implications. Indeed, a few reports explored the role of intronic or intergenic lncG clusters co-expressed and functionally synergistic in brain development [46] and cancer settings [45], [52], respectively. In this view, clustering can be interpreted as an opportunity for lncRNA coordinated expression, combinatorial activity and clinical significance in physiological and diseased mechanisms. Alternatively, lncG clustering may function as an epigenetic mark which may contribute to transcriptional accessibility or chromatin silencing, regulating gene expression on a broader scale, or maintaining local genomic stability. The interplay between lncG clustering and epigenetic control raises intriguing questions about whether these clusters serve as hubs for regulatory networks or scaffolds for chromatin architecture. The biological roles of most lncRNA clusters remain poorly understood [44], presenting a promising avenue for future research.

Another aspect of lncG that we have analyzed is related to their lengths, which appear mostly related to chromosome size. Discrepancies exist particularly in chromosomes 21 and Y, where lengths are longer than expected, and in chromosome 1, with shorter lengths than expected. Whether the size of lncGs is a regulative determinant for the functionality of the lncRNA mature form is a plausible factor that needs further exploration, as a significant portion of the genetic material was predicted to be spliced out after transcription. While we do not provide a detailed analysis of intron distribution or composition in lncGs, their impact can be even more important than in PCGs. For instance, their retention has been shown to contribute to new sequence modules at the lncRNA compartmentalization [14] or interaction with regulatory proteins [53]. This is in line with the observation that inefficient splicing of long non-coding RNAs is associated with higher transcript complexity in humans and mice [7].

Making a comparison with coding transcripts, we also inspected some functional elements within lncG sequences that could potentially affect their transcription. These elements, include a distinct pattern of GC content, that was particularly evident when comparing the 3’ and 5’ ends of these genes.

Finally, we examined the number and quality of nucleotide variations affecting lncRNA sequences. While human genetics studies predominantly focus on coding genes, often associated with distinct genetic or multifactorial diseases, we identified a number of clinically relevant SNPs impacting lncGs. It is important to note that some of these variations could affect antisense coding genes or regulatory regions relevant to coding genes. However, nowadays, there are several examples of SNPs affecting specific lncRNA transcripts that have been linked to a variety of human diseases [5]. As such, it is conceivable to hypothesize a role for the lncRNA-affecting SNPs identified in this work in a pathological context. In this view, the study of expression quantitative trait loci (eQTL), such as that conducted by the FANTOM project [24], [50], represents an invaluable source of information for understanding the implication of lncRNA nucleotide variations in genetically determined conditions and phenotypes.

## 4 Concluding remarks

This work provides an essential update on the organization, distribution, and structural characteristics of lncRNA genes. To the best of our knowledge, it represents one of the few comprehensive analyses of lncRNA genomic features in recent years. While our study offers a detailed and quantitative overview of lncRNA genomics, some limitations must be acknowledged. For instance, we do not address certain domain properties, such as DNA-, RNA-, and protein-binding features, which are critical for understanding lncRNA functionality. Additionally, while analyzing critical primary sequence determinants was valuable, the secondary structures formed by lncRNAs enable them to act as scaffolding molecules for various molecular partners [39]. These structural characteristics are highly context-dependent, relying on specific cellular environments, and require a study beyond the scope of this work.

Furthermore, our study highlights the current lack of comprehensive annotations for several lncRNA attributes, including subcellular localization, interaction networks, and mechanistic roles. These gaps present significant challenges in fully understanding their biological activity and improving computational prediction tools for this transcript class. Persistent issues with classification and nomenclature remain weak points in lncRNA research. Despite these challenges, this study presents a comprehensive virtual lncRNA karyotype that aims to serve as a foundational reference for future investigations. It provides insights into the functional landscape of the dark genome, its regulatory roles (e.g., specific domains, binding motifs, and associated factors), and its potential applications as both a diagnostic and therapeutic target in biomedical research.

## 5 Methods

### 5.1 Datasets used in this study

Annotation files related to both human lncRNA (Long non-coding RNA gene annotation) and coding (Comprehensive gene annotation) genes were downloaded from GENCODE Release 46 (GRCh38.p14) in GTF format. Sequence file of long non-coding transcripts (Transcript sequences) was downloaded from GENCODE in FASTA format. Polyadenylation data of all human transcripts (PolyA feature annotation) was downloaded from GENCODE in GTF format. SNPs data was downloaded from Ensembl release-110 in VCF v4.1 format. All datasets and webservers used were accessed between July 2024 and January 2025, unless otherwise specified.

### 5.2 Data processing and analysis

Analyses have been performed in the R environment (R version 4.1.2 (2021–11–01)). GTF files were read and processed using *rtracklayer* package [30]. FASTA files were read and processed using *Biostrings* [35]. Other R packages used for the analysis are *biomaRt* for gene and transcripts ID conversion, *VariantAnnotation*, *GenomicRanges* and *seqinr* for sequence analyses (including GC content computation by sliding windows and SNPs analysis). The full list of R packages and their versions is provided within the deposited code.

#### 5.2.1 Peak distributions

Long non-coding and protein-coding genes distributions (peaks) were computed with a 1 Megabase window and reported as rainfall plots using the *circlize* library. In peak quantification, these are the regions where the gene density exceeds a certain threshold, indicating that they are areas of unusually high concentration of either lncGs or PCGs. The threshold was set at the 95th percentile of the gene density distribution (*quantile(density_lnc$density, 0.95)* for lncGs and *quantile(density_cod$density, 0.95)* for PCGs). This means that the top 5 % of density values are considered "TRUE peaks" and represented as triangles, while “FALSE peaks” are represented as circles.

#### 5.2.2 Genes and transcripts quantification

Gene quantification has been performed by counting annotated genes in GENCODE based on their gene ID, considering the annotation “gene” in the “gene type” column, hence excluding other annotation types (e.g. transcripts, exons, CDS). Transcript quantification has been performed by aggregating transcripts by gene ID and then counting the number of different transcript IDs for each gene.

#### 5.2.3 Polyadenylation and exons analysis

Genomic elements (polyadenylation and exons) were retrieved from the annotation files and processed accordingly to compute the statistics (filtering in all polyadenylated genes or exon). Exons were counted aggregating them by transcripts or genes. Polyadenylation sites and signals were retrieved from the annotation files, and the distance between polyadenylation sites and signals was retrieved using their annotated genomic coordinates.

#### 5.2.4 GC content analysis

GC content was calculated by summing the number of G and C nucleotides for each transcript sequence from FASTA files using in-house scripts. GC skew computation was performed by implementing a function that calculates the GC content on a window size of 10 nucleotides. The results are divided by 20 chunks for each transcript sequence and then the average GC content is calculated for each chunk.

#### 5.2.5 LncRNA classification

LncRNA subclassification was performed on each annotated gene name, as follows: gene names starting as “LINC” were classified as “Long Intergenic lncRNA”; gene names starting with “ENSG” were annotated as “Novel Transcripts”; gene names ending with “-HG”, “-AS*”, “-DT*”, “-IT*”, “-OT*” were annotated as “MicroRNA Hosting Genes”, “Antisense lncRNAs”, “Divergent Transcripts”, “Intronic Transcripts” and “Overlapping Transcripts”, respectively. All the other genes were annotated as “Name-assigned lncRNAs”. As novel transcripts and name-assigned lncRNAs could also fall under other categories, they were excluded from the GC content analyses performed on lnRNA subclasses or grouped together under the category “Others”.

#### 5.2.6 LncRNA localization analysis

Localization of lncRNAs was determined by querying the "RNAlocate" database (http://www.rna-society.org/rnalocate/) which contains annotations for 589 lncRNAs and their subcellular localizations (accessed in July 2024). Nuclear or cytoplasmic localization was assigned based on the following criteria: lncRNAs annotated as 'Nucleoplasm,' 'Nucleus,' 'Chromatin,' 'Nuclear,' 'Nuclear speckle,' or 'Paraspeckles in the nucleus' were classified as nuclear lncRNAs. Conversely, lncRNAs with annotations including 'Cytosol,' 'Cytoplasm,' 'Ribosome,' 'Ribosome-free cytosol,' 'Mitochondrion,' or 'Endoplasmic reticulum' were classified as cytoplasmic lncRNAs.

#### 5.2.7 SNP analysis

SNP analysis was conducted for each chromosome using SNP data from Ensembl Variation (accessed in July 2024). SNPs were filtered based on their overlap with genomic regions corresponding to lncG or PCG sequences by intersecting their genomic coordinates (SNP start and end sites) with those of lncGs (gene start and end sites). Clinically associated SNPs were obtained as VCF files from the Ensembl database (accessed in July 2024) and used to refine the SNP dataset, retaining only clinically relevant SNPs located within lncG regions.

Downstream analyses involved quantifying SNPs by category (e.g., transitions/transversions) and evaluating the distribution of SNPs across different lncRNA classes for each chromosome. Pathogenicity information was extracted from the clinical SNP VCF file, utilizing the columns “Benign,” “Likely_benign,” “VUS” (Variants of Uncertain Significance), “Likely_pathogenic,” and “Pathogenic,” which indicate TRUE/FALSE values for each SNP.

To analyze experimentally validated lncRNA SNPs, data from lncRNASNP v3 (https://gong_lab.hzau.edu.cn/lncRNASNP3/#!/, accessed in January 2025) were used. The dataset, obtained from the section titled “Experimentally validated lncRNA-associated disease lists for 3 species in the lncRNASNP database” (Homo sapiens), was cross-referenced with Ensembl SNP data based on SNP genomic coordinates (start/end sites). This approach ensured that only clinically associated and experimentally validated SNPs were retained for further analysis.

For the computation of the pathogenicity of clinically associated and experimentally validated SNPs for each chromosome a customized script has been run. Whenever a variant was classified in more than one pathogenicity class, variants were assigned to a single category based on priority: pathogenic > likely_pathogenic > VUS > likely_benign > benign.

## Abbreviations

eQTL, expression quantitative trait locus

lncRNA, long non-coding RNA

lncG, long non-coding gene

PCG, protein-coding gene

SEM, standard error mean

snRNA, small nuclear RNA

snoRNA, small nucleolar RNA

SNP, single-nucleotide polymorphism

SNV, single nucleotide variation

## Funding

This research was funded by: 1) Sapienza University (RM12117A5DE7A45B and RM123188F6B80CE4), to MB; 2) Consiglio Nazionale delle Ricerche-CNR (projects DBA.AD005.225-NUTRAGE-FOE2021 and DSB.AD006.371-InvAt-FOE2022), to PL; 3) European Union - NextGenerationEU: National Center for Gene Therapy and Drug based on RNA Technology, CN3 - code: CN00000041; National Recovery and Resilience Plan (NRRP) MUR – M4C2 – Action 1.4 - Call “Potenziamento strutture di ricerca e di campioni nazionali di R&S” (Spoke 3 “Neurodegeneration”, CUP: B83C22002870006, to MB and Spoke 6 “RNA Drug Development”, CUP B83C22002860006, to PL); 4) by the European Union – Next-GenerationEU – National Recovery and Resilience Plan (NRRP) – M4C2, INVESTMENT N. 1.1, Call “PRIN 2022” (project 2022BYB33L, CUP: B53D23016090006), to MB and PL; 5) by the European Union - Next-GenerationEU - National Recovery and Resilience Plan (NRRP) – M4C2, INVESTMENT N. 1.1, Call “PRIN 2022 PNRR” D.D.1409 of 14th Sep 2022 (project P2022FFEWN RNA2FUN, CUP: B53D23026140001), to MB.

## CRediT authorship contribution statement

**Palma Alessandro:** Writing – review & editing, Writing – original draft, Visualization, Validation, Supervision, Software, Resources, Project administration, Methodology, Investigation, Formal analysis, Data curation, Conceptualization. **Ballarino Monica:** Writing – review & editing, Visualization, Supervision, Resources, Project administration, Investigation, Funding acquisition, Conceptualization. **Buonaiuto Giulia:** Writing – review & editing, Visualization, Formal analysis, Data curation. **Laneve Pietro:** Writing – review & editing, Supervision, Project administration, Investigation, Funding acquisition, Conceptualization.

## Declaration of Competing Interest

The authors declare the following financial interests/personal relationships which may be considered as potential competing interests: Pietro Laneve reports financial support was provided by European Union. Monica Ballarino reports financial support was provided by European Union. If there are other authors, they declare that they have no known competing financial interests or personal relationships that could have appeared to influence the work reported in this paper.

## Data Availability

The datasets generated and/or analyzed during the current study are available in the following repositories: GTF file with lncRNA annotation, Sequence file in FASTA format of long non-coding transcripts, and Polyadenylation data of all human transcripts: GENCODE Release 46 (GRCh38.p14) (https://www.gencodegenes.org/human/release_46.html); SNPs data in VCF v4.1 format: Ensembl release-110 (http://www.ensembl.org/Homo_sapiens/Info/Index). The code used for the statistics has been deposited on Zenodo repository (record number: 14741786).

## Appendix A Supplementary material

Supplementary material

**Supplementary Fig. 1. (a)** Stacked barplot showing the percentages of PCGs and lncG across chromosomes. **(b)** Distribution of lncRNA lengths evidence the existence of three main peaks centered at ∼600, 800 and 1500 bp. **(c)** Distribution of the GC content for polyadenylated (PolyA^+^) and non-polyadenylated PolyA minus (PolyA^-^) cytoplasmic and nuclear long non-coding transcripts. **(d)** Stacked barplot reporting the number of genes belonging to each lncRNA subtype across the different chromosomes. **(e)** Distribution of the distance between polyA sites and signals for long non-coding (left) and coding (right) genes

## Footnotes

Appendix A Supplementary data associated with this article can be found in the online version at doi:10.1016/j.csbj.2025.01.026.

## References

[1] Abugessaisa I., Ramilowski J.A., Lizio M., Severin J., Hasegawa A., Harshbarger J. (2021). FANTOM enters 20th year: expansion of transcriptomic atlases and functional annotation of non-coding RNAs. Nucleic Acids Res.

[2] Amaral P., Carbonell-Sala S., De La Vega F.M., Faial T., Frankish A., Gingeras T. (2023). The status of the human gene catalogue. Nature.

[3] Anderson D.M., Anderson K.M., Chang C.L., Makarewich C.A., Nelson B.R., McAnally J.R. (2015). A micropeptide encoded by a putative long noncoding RNA regulates muscle performance. Cell.

[4] Ariel F., Lucero L., Christ A., Mammarella M.F., Jegu T., Veluchamy A. (2020). R-Loop mediated trans action of the APOLO long noncoding RNA. Mol Cell.

[5] Aznaourova M., Schmerer N., Schmeck B., Schulte L.N. (2020). Disease-causing mutations and rearrangements in long non-coding RNA gene loci. Front Genet.

[6] Ballarino M., Pepe G., Helmer-Citterich M., Palma A. (2023). Exploring the landscape of tools and resources for the analysis of long non-coding RNAs. Comput Struct Biotechnol J.

[7] Basu K., Dey A., Kiran M. (2023). Inefficient splicing of long non-coding RNAs is associated with higher transcript complexity in human and mouse. RNA Biol.

[8] Brown J.A., Valenstein M.L., Yario T.A., Tycowski K.T., Steitz J.A. (2012). Formation of triple-helical structures by the 3′-end sequences of MALAT1 and MENβ noncoding RNAs. Proc Natl Acad Sci.

[9] Cloutier S.C., Wang S., Ma W.K., Al Husini N., Dhoondia Z., Ansari A. (2016). Regulated formation of lncRNA-DNA hybrids enables faster transcriptional induction and environmental adaptation. Mol Cell.

[10] Cohen N., Dagan T., Stone L., Graur D. (2005). GC composition of the human genome: in search of isochores. Mol Biol Evol.

[11] Derrien T., Johnson R., Bussotti G., Tanzer A., Djebali S., Tilgner H. (2012). The GENCODE v7 catalog of human long noncoding RNAs: analysis of their gene structure, evolution, and expression. Genome Res.

[12] Desideri F., Cipriano A., Petrezselyova S., Buonaiuto G., Santini T., Kasparek P. (2020). Intronic determinants coordinate charme lncrna nuclear activity through the interaction with MATR3 and PTBP1. Cell Rep.

[13] Dey P., Mattick J.S. (2021). High frequency of intron retention and clustered H3K4me3-marked nucleosomes in short first introns of human long non-coding RNAs. Epigenetics Chromatin.

[14] Dumbović G., Braunschweig U., Langner H.K., Smallegan M., Biayna J., Hass E.P. (2021). Nuclear compartmentalization of TERT mRNA and TUG1 lncRNA is driven by intron retention. Nat Commun.

[15] Esposito R., Lanzós A., Uroda T., Ramnarayanan S., Büchi I., Polidori T. (2023). Tumour mutations in long noncoding RNAs enhance cell fitness. Nat Commun.

[16] Frankish A., Carbonell-Sala S., Diekhans M., Jungreis I., Loveland J.E., Mudge J.M. (2023). GENCODE: reference annotation for the human and mouse genomes in 2023. Nucleic Acids Res.

[17] Gibbons H.R., Shaginurova G., Kim L.C., Chapman N., Spurlock C.F., Aune T.M. (2018). Divergent lncRNA GATA3-AS1 Regulates GATA3 Transcription in T-Helper 2 Cells. Front Immunol.

[18] Ginno P., Lott P., Christensen H., Korf I., and F., C. (2012). R-loop formation is a distinctive characteristic of unmethylated human CpG island promoters. Mol Cell.

[19] Grimwood J., Gordon L.A., Olsen A., Terry A., Schmutz J., Lamerdin J. (2004). The DNA sequence and biology of human chromosome 19. Nature.

[20] Haerty W., Ponting C.P. (2015). Unexpected selection to retain high GC content and splicing enhancers within exons of multiexonic IncRNA loci. Rna.

[21] Haraksingh R.R., Snyder M.P. (2013). Impacts of variation in the human genome on gene regulation. J Mol Biol.

[22] Hartford C.C.R., Lal A. (2020). When long noncoding becomes protein coding. Mol Cell Biol.

[23] Hartono S.R., Korf I.F., Chédin F. (2015). GC skew is a conserved property of unmethylated CpG island promoters across vertebrates. Nucleic Acids Res.

[24] Hon C.-C., Ramilowski J.A., Harshbarger J., Bertin N., Rackham O.J.L., Gough J. (2017). An atlas of human long non-coding RNAs with accurate 5′ ends. Nature.

[25] Illingworth R.S., Bird A.P. (2009). CpG islands - a rough guide. FEBS Lett.

[26] Jdo J.W.I., Baldini A., Ward D.C., Reeders S., Wells R.A. (1991). Origin of human chromosome 2: an ancestral telomere-telomere fusion. Proc Natl Acad Sci.

[27] Karczewski K.J., Francioli L.C., Tiao G., Cummings B.B., Alföldi J., Wang Q. (2020). The mutational constraint spectrum quantified from variation in 141,456 humans. Nature.

[28] Lander E.S., Linton L.M., Birren B., Nusbaum C., Zody M.C., Baldwin J. (2001). Initial sequencing and analysis of the human genome: international human genome sequencing consortium (Nature (2001) 409 (860-921)). Nature.

[29] Laurent G.S., Wahlestedt C., Kapranov P. (2015). The landscape of long non-coding RNA classification. Trends Genet.

[30] Lawrence M., Gentleman R., Carey V. (2009). rtracklayer: an R package for interfacing with genome browsers. Bioinformatics.

[31] Markaki Y., Gan Chong J., Wang Y., Jacobson E.C., Luong C., Tan S.Y.X. (2021). Xist nucleates local protein gradients to propagate silencing across the X chromosome. Cell.

[32] Martin F.J., Amode M.R., Aneja A., Austine-Orimoloye O., Azov A.G., Barnes I. (2023). Ensembl 2023. Nucleic Acids Res.

[33] Mattick J.S., Amaral P.P., Carninci P., Carpenter S., Chang H.Y., Chen L.L. (2023). Long non-coding RNAs: definitions, functions, challenges and recommendations. Nat Rev Mol Cell Biol.

[34] Niehrs C., Luke B. (2020). Regulatory R-loops as facilitators of gene expression and genome stability. Nat Rev Mol Cell Biol.

[35] Pagès, H., Aboyoun, P., Gentleman, R., and DebRoy, S. (2021). Biostrings: Efficient manipulation of biological strings.

[36] Pan J., Wang R., Shang F., Ma R., Rong Y., Zhang Y. (2022). Functional micropeptides encoded by long non-coding RNAs: a comprehensive review. Front Mol Biosci.

[37] Piovesan A., Pelleri M.C., Antonaros F., Strippoli P., Caracausi M., Vitale L. (2019). On the length, weight and GC content of the human genome. BMC Res Notes.

[38] Qiu Y., Kang Y.M., Korfmann C., Pouyet F., Eckford A., Palazzo A.F. (2024). The GC-content at the 5′ ends of human protein-coding genes is undergoing mutational decay. Genome Biol.

[39] Ribeiro D.M., Zanzoni A., Cipriano A., Ponti R.D., Spinelli L., Ballarino M. (2018). Protein complex scaffolding predicted as a prevalent function of long non-coding RNAs. Nucleic Acids Res.

[40] Schlackow M., Nojima T., Gomes T., Dhir A., Carmo-Fonseca M., Proudfoot N.J. (2017). Distinctive patterns of transcription and RNA processing for human lincRNAs. Mol Cell.

[41] Seal R.L., Chen L., Griffiths-Jones S., Lowe T.M., Mathews M.B., O’Reilly D. (2020). A guide to naming human non-coding RNA genes. EMBO J.

[42] Sherry S.T., Ward M.H., Kholodov M., Baker J., Phan L., Smigielski E.M. (2001). DbSNP: the NCBI database of genetic variation. Nucleic Acids Res.

[43] Statello L., Guo C.J., Chen L.L., Huarte M. (2021). Gene regulation by long non-coding RNAs and its biological functions. Nat Rev Mol Cell Biol.

[44] Sun J., Zhou M., Mao Z.T., Hao D.P., Wang Z.Z., Li C.X. (2013). Systematic analysis of genomic organization and structure of long non-coding RNAs in the human genome. FEBS Lett.

[45] Tomita S., Abdalla M.O.A., Fujiwara S., Matsumori H., Maehara K., Ohkawa Y. (2015). A cluster of noncoding RNAs activates the ESR1 locus during breast cancer adaptation. Nat Commun.

[46] Wang F., Ren D., Liang X., Ke S., Zhang B., Hu B. (2019). A long noncoding RNA cluster-based genomic locus maintains proper development and visual function. Nucleic Acids Res.

[47] Wilusz J.E., JnBaptiste C.K., Lu L.Y., Kuhn C.D., Joshua-Tor L., Sharp P.A. (2012). A triple helix stabilizes the 3′ ends of long noncoding RNAs that lack poly(A) tails. Genes Dev.

[48] Yao R.W., Wang Y., Chen L.L. (2019). Cellular functions of long noncoding RNAs. Nat Cell Biol.

[49] Yin Q.F., Yang L., Zhang Y., Xiang J.F., Wu Y.W., Carmichael G.G. (2012). Long Noncoding RNAs with snoRNA Ends. Mol Cell.

[50] Yip C.W., Hon C.C., Yasuzawa K., Sivaraman D.M., Ramilowski J.A., Shibayama Y. (2022). Antisense-oligonucleotide-mediated perturbation of long non-coding RNA reveals functional features in stem cells and across cell types. Cell Rep.

[51] Zhen N., Zhu J., Mao S., Zhang Q., Gu S., Ma J. (2023). Alternative splicing of lncRNAs From SNHG family alters snoRNA expression and induces chemoresistance in hepatoblastoma. Cmgh.

[52] Zhou H., Gao Y., Li X., Shang S., Wang P., Zhi H. (2021). Identifying and characterizing lincRNA genomic clusters reveals its cooperative functions in human cancer. J Transl Med.

[53] Zhou H.Z., Li F., Cheng S.T., Xu Y., Deng H.J., Gu D.Y. (2022). DDX17-regulated alternative splicing that produced an oncogenic isoform of PXN-AS1 to promote HCC metastasis. Hepatology.

[54] Zhou V.W., Goren A., Bernstein B.E. (2011). Charting histone modifications and the functional organization of mammalian genomes. Nat Rev Genet.

