# Supplementary figures and images for: Genome biology of long non-coding RNAs in humans: A virtual karyotype
Source: Comput Struct Biotechnol J. 2025 Jan 31;27:575–84. doi: 10.1016/j.csbj.2025.01.026 (PMC11847481; doi:10.1016/j.csbj.2025.01.026)

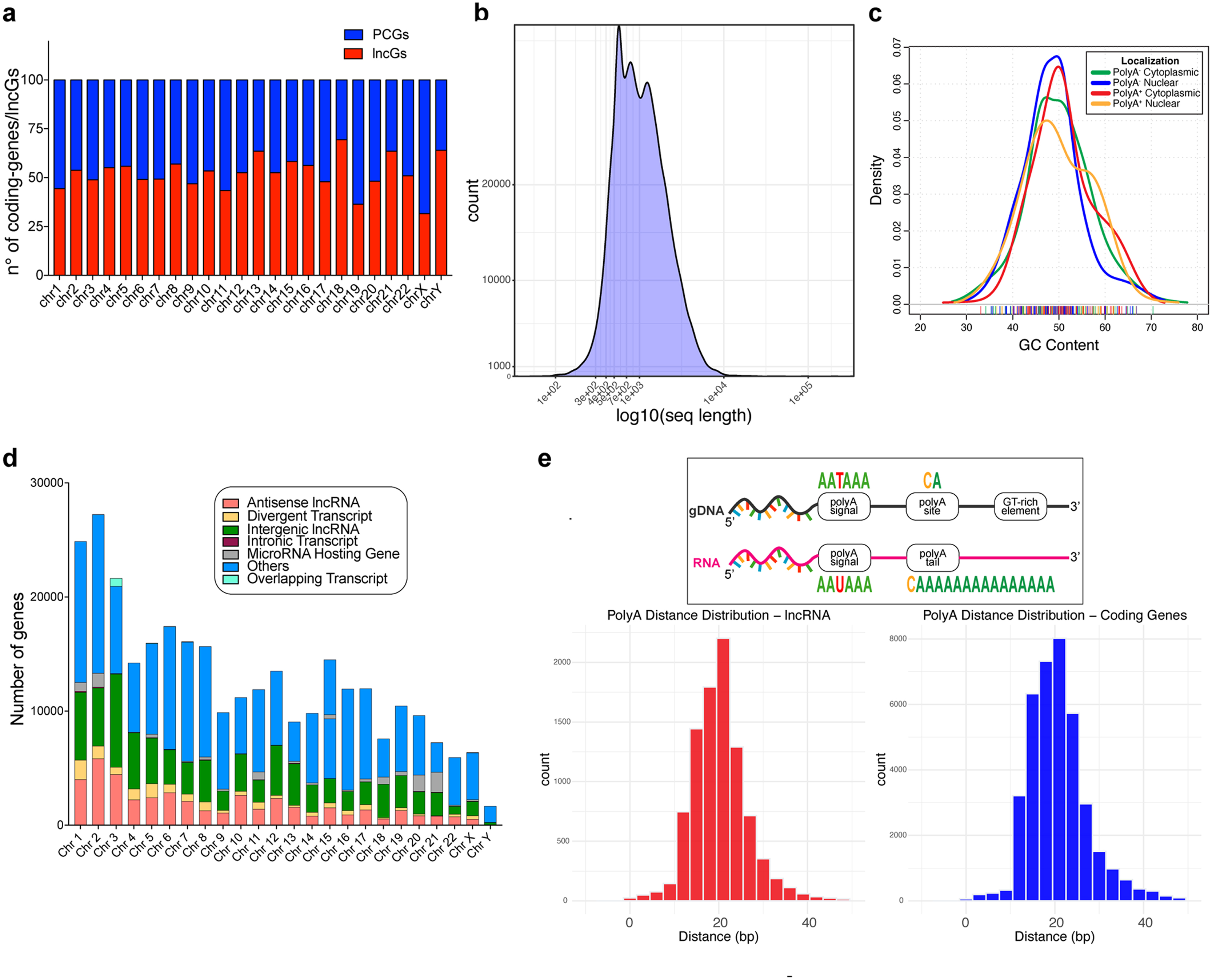

Supplement: Supplementary file 4 — Supplementary Fig. 1. (a) Stacked barplot showing the percentages of PCGs and lncG across chromosomes. (b) Distribution of lncRNA lengths evidence the existence of three main peaks centered at ∼600, 800 and 1500 bp. (c) Distribution of the GC content for polyadenylated (PolyA+) and non-polyadenylated PolyA minus (PolyA-) cytoplasmic and nuclear long non-coding transcripts. (d) Stacked barplot reporting the number of genes belonging to each lncRNA subtype across the different chromosomes. (e) Distribution of the distance between polyA sites and signals for long non-coding (left) and coding (right) genes [file mmc4.jpg]
